# Ursodeoxycholic acid in intrahepatic cholestasis of pregnancy: a secondary analysis of the PITCHES trial

**DOI:** 10.1111/1471-0528.16567

**Published:** 2020-11-08

**Authors:** J Fleminger, PT Seed, A Smith, E Juszczak, PH Dixon, J Chambers, J Dorling, C Williamson, JG Thornton, LC Chappell

**Affiliations:** ^1^ Department of Women and Children’s Health School of Life Course Sciences King’s College London London UK; ^2^ National Perinatal Epidemiology Unit Clinical Trials Unit Nuffield Department of Population Health University of Oxford Oxford UK; ^3^ ICP Support Sutton Coldfield UK; ^4^ Division of Neonatal‐Perinatal Medicine IWK Health Centre Halifax NS Canada; ^5^ Division of Child Health, Obstetrics and Gynaecology University of Nottingham Nottingham UK

**Keywords:** Cholestasis, perinatal, pregnancy, stillbirth, ursodeoxycholic acid

## Abstract

**Objective:**

To evaluate whether a particular group of women with intrahepatic cholestasis of pregnancy (ICP), based on their presenting characteristics, would benefit from treatment with ursodeoxycholic acid (UDCA).

**Design:**

Secondary analysis of the PITCHES trial (ISRCTN91918806).

**Setting:**

United Kingdom.

**Population or Sample:**

527 women with ICP.

**Methods:**

Subgroup analyses were performed to determine whether baseline bile acid concentrations or baseline itch scores moderated a woman’s response to treatment with UDCA.

**Main outcome measures:**

Bile acid concentration and itch score.

**Results:**

In women with baseline bile acid concentrations less than 40 μmol/l, treatment with UDCA resulted in increased post‐randomisation bile acid concentrations (geometric mean ratio 1.19, 95% CI 1.00–1.41, *P* = 0.048). A test of interaction showed no significance (*P* = 0.647). A small, clinically insignificant difference was seen in itch response in women with a high baseline itch score (–6.0 mm, 95% CI −11.80 to −0.21, *P* = 0.042), with a test of interaction not showing significance (*P* = 0.640). Further subgroup analyses showed no significance. Across all women there was a weak relationship between bile acid concentrations and itch severity.

**Conclusions:**

There was no subgroup of women with ICP in whom a beneficial effect of treatment with UDCA on bile acid concentration or itch score could be identified. This confirms that its routine use in women with this condition for improvement of bile acid concentration or itch score should be reconsidered.

**Tweetable abstract:**

PITCHES: No group of women with ICP has been found in whom UDCA reduces bile acid concentrations or pruritus.

## Tweetable abstract

PITCHES: No group of women with ICP has been found in whom UDCA reduces bile acid concentrations or pruritus.

## Introduction

Intrahepatic cholestasis of pregnancy (ICP) is characterised by maternal pruritus and elevated serum bile acids. It affects around 0.7% of pregnancies in the UK^1^ and typically presents in the third trimester. It is associated with adverse perinatal outcomes including stillbirth, preterm labour and neonatal unit admission. An increase in preterm birth is seen with serum bile acid concentrations above 40 µmol/l, and the risk of stillbirth is increased in women when peak serum bile acid concentrations are 100 µmol/l or more.^2^

The mainstay of treatment has been ursodeoxycholic acid (UDCA), a bile acid produced in small amounts by the gut microbiota in humans.^3^ Surveys across the UK found that 97% of obstetricians used UDCA to treat ICP^4^ and it is recommended in six national guidelines, but evidence for its efficacy is limited. A 2013 Cochrane review^5^ of 21 trials with a total of 1197 women concluded that UDCA improved maternal pruritus, albeit by a small amount. In women treated with UDCA there was no difference in adverse perinatal outcomes, but the number of events overall was small. The largest trial analysed in that meta‐analysis included only 111 women, and the majority of the trials were assessed as being at moderate‐to‐high risk of bias.

The PITCHES trial was published in August 2019 and was the largest trial to date investigating the efficacy of UDCA in 605 women with ICP.^6^ It was a parallel‐group, double‐blind, multicentre, randomised placebo‐controlled trial with individual randomisation to UDCA or placebo using a 1:1 allocation ratio. The primary outcome of the trial was a composite measure of adverse perinatal outcomes. Secondary maternal data, including biochemical measurements and itch severity, were collected at antenatal visits from randomisation to delivery. The trial found that there was no evidence that treatment with UDCA significantly reduced adverse perinatal outcomes.

It is nevertheless possible that there is a group of women with ICP who do respond to treatment with UDCA, either in terms of a reduction in bile acid concentrations or in itch severity, possibly leading to better perinatal outcomes or symptomatic relief. These women would need to be identified from their presenting characteristics in order for them to receive targeted treatment. This secondary analysis of the PITCHES trial aims to investigate whether a particular group of women, identified by their characteristics at presentation, might benefit from targeted treatment with UDCA.

## Methods

This is a secondary analysis of the PITCHES study, a parallel‐group, double‐blind, multicentre, randomised placebo‐controlled trial with individual randomisation to UDCA or placebo using a 1:1 allocation ratio. The original primary analysis was published in August 2019.^6^ A prespecified statistical analysis plan was written for this secondary analysis (Appendix S1). ICP Support, the patient support charity, were involved in the design of the secondary analysis to ensure that clinically relevant outcomes were studied.

Women were eligible to enrol in the trial if they had a diagnosis of intrahepatic cholestasis of pregnancy, characterised by pruritus and an increase in serum bile acid concentration above the upper limit of normal in their local laboratory. Additional inclusion criteria included: being between 20 weeks and 0 days, and 40 weeks and 6 days of pregnancy on the day of randomisation, a singleton or twin pregnancy, no known lethal fetal anomaly, aged 18 years or over, and able to give written informed consent. Participants were randomly allocated to receive UDCA or placebo using a 1:1 ratio, using a minimisation algorithm. Trial participants, clinical care providers, outcome assessors and data analysis were all masked to allocation. The placebo and UDCA tablets looked identical. A starting dose of two tablets a day was recommended (equivalent to a UDCA dose of 500 mg twice a day in the UDCA group). This dose could be increased or decreased by one tablet a day every 3–14 days up to a maximum of four tablets a day, at a clinician's discretion. It was recommended that treatment should continue from enrolment until the infant’s birth.

The primary outcome was a composite of perinatal death (defined as in‐utero fetal death after randomisation or known neonatal death up to 7 days after birth), preterm delivery (<37 weeks’ gestation), or neonatal unit admission for at least 4 hours (from birth until hospital discharge). Each infant was counted once within this composite. Secondary maternal outcomes were collected on all women at clinical visits between randomisation and delivery. These included serum bile acid concentration (µmol/l) and itch severity (measured as the worst episode of itch over the past 24 hours in mm on a 0–100 mm visual analogue scale, where 100 mm was the worst itch). Secondary perinatal outcomes were collected on case‐note review after infant discharge. Full details about the original trial can be found in the protocol and primary analysis.^6^, ^7^

### Maternal outcomes

All analyses in this secondary analysis followed the intention‐to‐treat principle: all randomly allocated women were analysed according to the group they were allocated to, irrespective of the treatment they received, if any. The analyses required data collected at post‐randomisation visits therefore women without post‐randomisation visit data were excluded.

For bile acid concentrations, subgroups were defined based on accepted thresholds from the literature related to perinatal risk.^2^ For itch, subgroups were defined based on median itch at baseline in the trial participants.^6^ The effect of baseline bile acid concentration (<40 μmol/l versus ≥40 μmol/l, <100 μmol/l versus ≥100 μmol/l) and baseline itch score (<60 mm versus ≥60 mm) on two maternal outcomes were analysed: (1) serum bile acid concentration post‐randomisation and (2) itch score post‐randomisation. Baseline bile acid trajectory was defined as ‘increasing’ if the first bile acid concentration post‐randomisation was greater than or equal to the baseline bile acid concentration and ‘decreasing’ if the first bile acid concentration post‐randomisation was less than the baseline bile acid concentration. The effect of this baseline itch trajectory on the two maternal outcomes was also analysed.

As bile acid concentrations demonstrate a lognormal distribution, the geometric mean of all available post‐randomisation bile acid concentrations was used to calculate each participant’s mean post‐randomisation bile acid concentration, and the trial groups were compared using a geometric mean ratio. An arithmetic mean of all available itch scores post‐randomisation was used to calculate each participant’s mean post‐randomisation itch score, and the trial groups were compared using a mean difference. An interaction test (likelihood ratio version) was used to test for a difference in treatment effect between the individual subgroups. Interaction tests compare the goodness‐of‐fit of two models: one including the subgroups in question and one excluding them. If the model including the subgroups is better at representing the underlying data (concluded if the output is statistically significant) then there is justification for targeting the underlying subgroup. If the model including the subgroups is not better at representing the underlying data (concluded if the output is not statistically significant) then there is no justification for targeting the underlying subgroup, irrespective of whether the subgroup itself demonstrates statistical significance.

The treatment effect in each group was visualised by plotting the average itch score or geometric average bile acid concentration by visit. Visits were seven days apart, plus or minus one day. Only visits which had five or more women contributing results were included. Error bars were added to represent the standard error of each group at each visit. All available pairs of itch scores and bile acid concentrations were plotted for all participants at all time points, and a correlation coefficient between the two was calculated.

### Repeat survey of minimal clinically important difference

Two surveys that were originally carried out for the PITCH pilot study^8^ were repeated in order to re‐evaluate their findings in current times. These surveys were created to determine the minimal clinically important difference (MCID) in itch score. The two surveys (one designed for women and one designed for clinicians) were completed using the online survey platform SurveyMonkey. The survey for clinicians was disseminated by the authors by email, via mailing lists of local and national obstetric medicine groups, and by Twitter and Facebook. The survey for women was disseminated by ICP Support, the patient support charity, through their social media channels.

Each survey asked two questions. The first question directly replicated a question in the original survey. Women and clinicians were told that the mean baseline itch score on a visual analogue scale of 0 to 100 mm was 60 mm. They were then asked to identify from a choice of distances on the scale, what size reduction in itch score they considered clinically meaningful. The second question asked the participant to identify the proportion of women who would need to change from itching so severe that they were unable to sleep through the night, to being able to sleep through the night, before they would consider taking or prescribing a drug. The full questions can be found in Appendix S2. Each survey also asked questions about basic demographics. The survey for women asked whether they had personal experience of ICP.

Basic demographics for both clinicians and women were calculated. Only women with prior experience with ICP were included in the analysis. The median value and interquartile range for each question in each survey was calculated. Responses were compared between women with and without prior experience of ICP.

All calculations in this secondary analysis were performed in Stata version 17 and replicated in R. All graphical outputs were created in R.

### Funding

The trial was funded by the National Institute for Health Research Efficacy and Mechanism Evaluation Programme (Reference 12/164/16), following external peer review, including patient and public review.

## Results

Between 23rd December 2015 and 7th August 2018, 2737 women were screened for trial inclusion, of whom 1418 were found to be eligible. 605 women were recruited to the trial, including 37 women with a twin pregnancy, across 33 maternity units. 17 of the 33 maternity units used a threshold of 14 μmol/l as the upper limit of normal for serum bile acid concentration, whereas the remaining units used thresholds between 10 and 13 μmol/l, according to local laboratory reference ranges. 305 women were randomly allocated to UDCA and 300 women were randomly allocated to placebo. Follow‐up to maternal and infant discharge continued until December 2018.^6^

Of the 605 women recruited, 76 women with no post‐randomisation visit data were excluded from the secondary analysis. The majority of these women (73/76, 96%) delivered within 2 weeks of randomisation. Two women withdrew from the trial: one withdrew consent for further data collection and no post‐randomisation data was collected, and one withdrew consent to use data. Both were also excluded. Maternal baseline characteristics and maternal outcomes for the 527 women included in the secondary analysis (256 allocated to placebo, 271 allocated to UDCA) are shown in Table S1. Perinatal outcomes for the 558 infants born to these women are shown in Table S2. A flow diagram describing the participants included in each individual analysis is shown in Figure S1.

### Maternal outcomes

Bile acid concentrations and itch scores post‐randomisation, stratified by baseline characteristics, are shown in Table 1.

**Table 1:** Bile acid concentration and itch score post‐randomisation, stratified by baseline characteristics

	UDCA (*n* = 271)	Placebo (*n* = 256)	Adjusted effects estimate (95% CI), *P* value	Interaction test (*P*)
Bile acid concentration, all women, *N*	256	247		
Bile acid concentration (μmol/l), geometric mean (SD)	22.8 (2.4)	19.0 (2.8)	GMR 1.17 (1.00 to 1.36) 0.045	
Baseline bile acid conc. <40 μmol/l, *N*	196	191		0.647
Bile acid concentration (μmol/l), geometric mean (SD)	19.1 (2.3)	16.0 (2.6)	GMR 1.19 (1.00 to 1.41) 0.047	
Baseline bile acid conc. ≥40 μmol/l, *N*	60	56		
Bile acid concentration (μmol/l), geometric mean (SD)	40.6 (2.2)	34.5 (2.9)	GMR 1.13 (0.81 to 1.58) 0.466	
Baseline bile acid conc. <100 μmol/l, *N*	236	233		0.458
Bile acid concentration (μmol/l), geometric mean (SD)	21.3 (2.3)	17.8 (2.7)	GMR 1.19 (1.02 to 1.39) 0.031	
Baseline bile acid conc. ≥100 μmol/l, *N*	20	14		
Bile acid concentration (μmol/l), geometric mean (SD)	49.6 (2.5)	56.8 (3.2)	GMR 0.97 (0.48 to 1.96) 0.941	
Itch score, all women, *N*	241	227		
Itch score (mm), mean (SD)	49.8 (25.8)	56.6 (26.8)	MD −5.26 (−9.48 to −1.03) 0.015	
Baseline itch score <60 mm, *N*	108	90		0.640
Itch score (mm), mean (SD)	39.9 (23.9)	43.3 (23.6)	MD −4.04 (−10.33 to 2.26) 0.211	
Baseline itch score ≥60 mm, *N*	133	137		
Itch score (mm), mean (SD)	57.9 (24.6)	65.4 (25.1)	MD −6.00 (−11.73 to −0.28) 0.041	
Baseline bile acid conc. <40 μmol/l, *N*	187	178		0.455
Itch score (mm), mean (SD)	49.6 (25.6)	54.9 (26.5)	MD −4.41 (−9.13 to 0.30) 0.067	
Baseline bile acid conc. ≥40 μmol/l, *N*	54	49		
Itch score (mm), mean (SD)	50.4 (26.8)	62.8 (27.1)	MD −8.03 (−17.68 to 1.62) 0.106	
Baseline bile acid conc. <100 μmol/l, *N*	226	216		0.753
Itch score (mm), mean (SD)	49.9 (25.6)	56.3 (26.5)	MD −5.37 (−9.63 to −1.10) 0.014	
Baseline bile acid conc. ≥100 μmol/l, *N*	15	11		
Itch score (mm), mean (SD)	48.7 (29.6)	63.1 (31.8)	MD −5.77 (−34.12 to 22.57) 0.693	

Adjusted effects estimates adjusted for baseline values.

GMR, Geometric Mean Ratio; MD, Mean Difference; SD, standard deviation.

**Figure 1:**
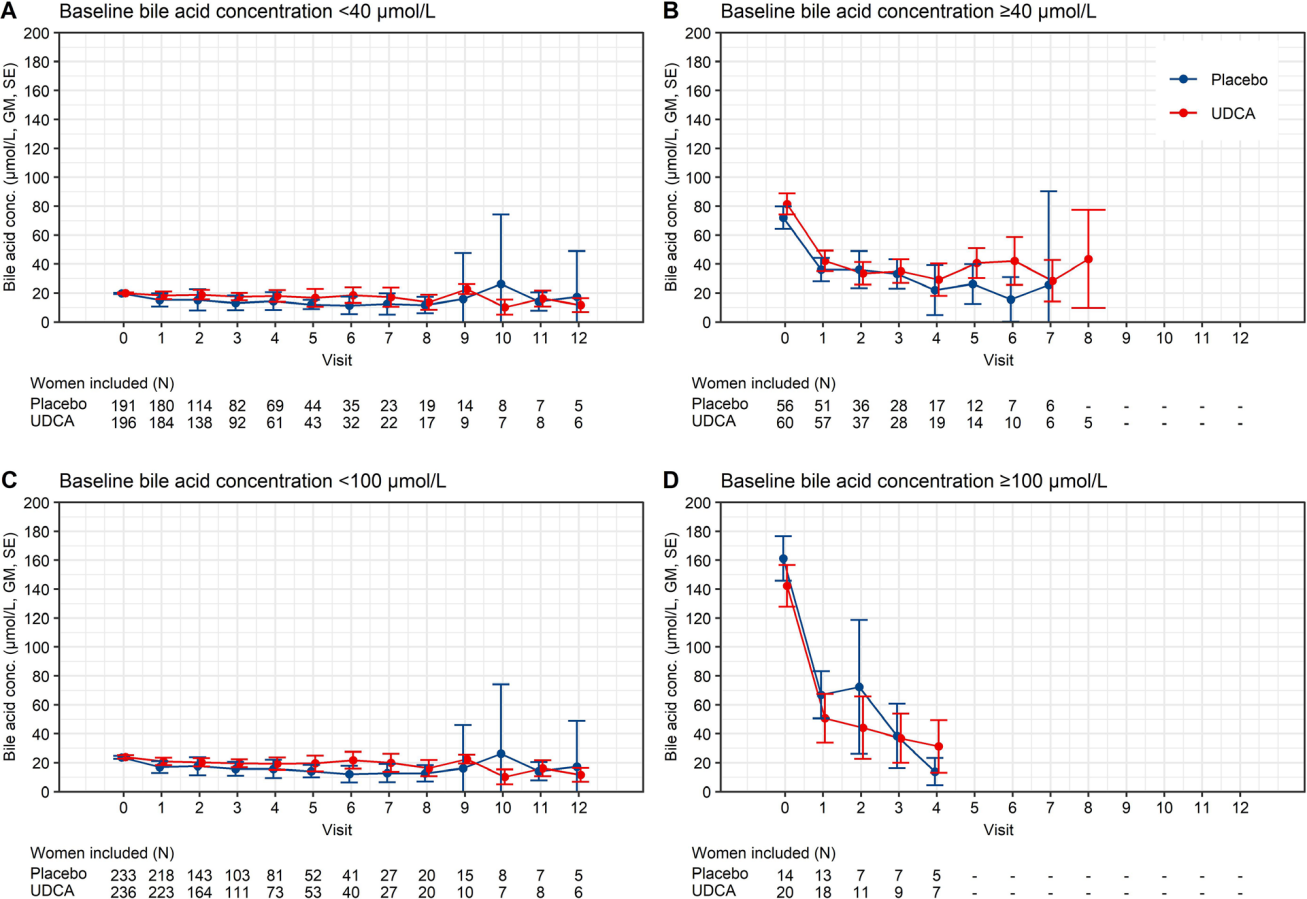
Bile acid concentration mean trajectories stratified by baseline characteristics. Changes in bile acid concentration by visit post‐randomisation, stratified by baseline bile acid concentration <40 μmol/l (A) versus ≥40 μmol/l (B), and by baseline bile acid concentration <100 μmol/l (C) versus ≥100 μmol/l (D). Numbers below each graph refer to number of women contributing results at each point. Only visits with five or more women were included. Visit 0 = baseline visit. GM = geometric mean, SE = standard error.

Mean trajectories of bile acid concentration stratified by baseline bile acid concentration are shown in Figure 1. Visual inspection shows that bile acid concentrations reduced in women taking UDCA and placebo, most notable in women with baseline bile acid concentrations ≥40 μmol/l (Figure 1B), and also seen in women with baseline bile acid concentrations ≥100 μmol/l (Figure 1D). In women with baseline bile acid concentrations <40 μmol/l (Figure 1A), treatment with UDCA resulted in higher post‐randomisation bile acid concentrations compared to placebo (geometric mean ratio 1.19, 95% CI 1.00–1.41, *P* = 0.047). There was no significant increase in women with higher baseline bile acid concentrations (≥40 μmol/l, Figure 1B) (geometric mean ratio 1.13, 95% CI 0.81–1.58, *P* = 0.466). A test of interaction showed no significance (*P* = 0.647). When stratified by a higher threshold (100 μmol/l), in women with baseline bile acid concentrations <100 μmol/l (Figure 1C), treatment with UDCA resulted in increased post‐randomisation bile acid concentrations (geometric mean ratio 1.19, 95% CI 1.02–1.39, *P* = 0.031). There was no significant difference in women with baseline bile acid concentrations ≥100 μmol/l (Figure 1D) (geometric mean ratio 0.97, 95% CI 0.48–1.96, *P* = 0.941). A test of interaction showed no significance (*P* = 0.458).

**Figure 2:**
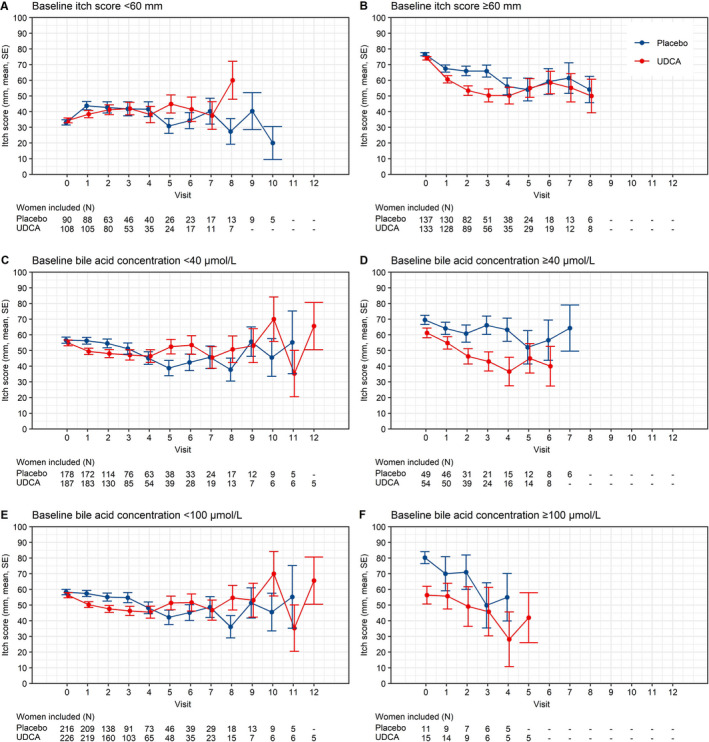
Itch score mean trajectories stratified by baseline characteristics. Changes in itch score by visit post‐randomisation, stratified by baseline itch score (A and B: <60 mm versus ≥60 mm) and by bile acid concentration (C and D: <40 μmol/l versus ≥40 μmol/l, E and F: <100 μmol/l versus ≥100 μmol/l). Numbers below each graph refer to number of women contributing results at each point. Only visits with five or more women were included. Visit 0 = baseline visit. GM = geometric mean, SE = standard error.

Mean trajectories of itch score stratified by baseline itch score and baseline bile acid concentration are shown in Figure 2. In women with a high baseline itch score (≥60 mm, Figure 2B), treatment with UDCA resulted in a small 6.0 mm decrease in itch score compared to placebo (*P* = 0.041). There was no evidence of a significant effect of UDCA seen in women with a baseline itch score <60 mm (Figure 2A). A test of interaction showed no significance (*P* = 0.640). Although the post‐randomisation itch score trajectories appear to be different at certain individual visits for women with higher baseline bile acid concentrations (Figure 2D), the mean difference in itch score post‐randomisation over gestation was not significantly different between the groups (−8.03 mm, 95% CI −17.68 to 1.62 mm, *P* = 0.106). The test of interaction was also not significant (*P* = 0.455), confirming that there was no subgroup stratified by a baseline bile acid concentration of 40 μmol/l in whom itch trajectory was different. Similarly, a test of interaction showed no significance (*P* = 0.753) in women stratified by a baseline bile acid concentration threshold of 100 μmol/l (Figure 2E,F). The results of these subgroup analyses were confirmed by a sensitivity analysis restricted to women taking >90% of medications (by self‐report) (Table S4).

Mean trajectories of bile acid concentration and itch score were further examined, stratified by initial bile acid trajectory either increasing or decreasing, as shown in Figures S2 and S3. Tests of interaction showed no significance for moderation of either post‐randomisation bile acid concentrations or post‐randomisation itch score.

**Figure 3:**
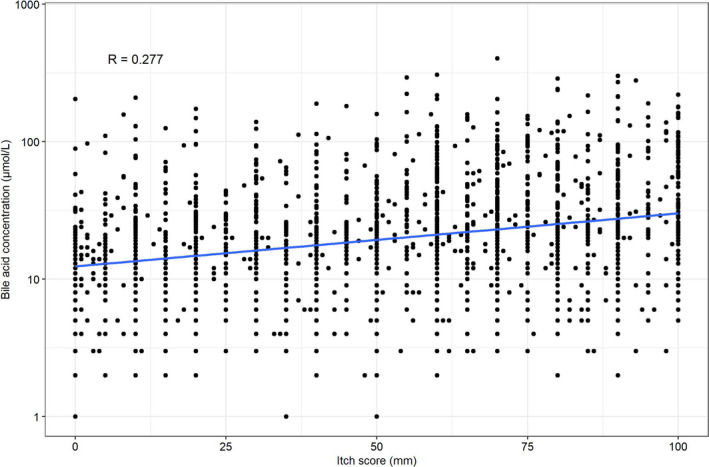
Relationship between itch score and bile acid concentration. Scatter plot of itch score versus bile acid concentration, for all women at all available visits. Bile acid concentration plotted on logarithmic scale.

The relationship between itch score and bile acid concentration for all women at all visits is shown in Figure 3. The correlation coefficient (R) was 0.277.

### Repeat survey of minimal clinically important difference

Between 24th January and 5th March 2020, 650 women completed our survey via ICP Support’s social media channels. 450 (73%) had current or prior experience of ICP. 116 clinicians completed the survey. The demographics of each group can be found in Table S3.

Clinicians indicated that they considered that a 30 mm (median, IQR 20–30 mm) improvement in the visual analogue scale score (from a baseline score of 60 mm) would be a clinically important difference, as did the women with prior experience of ICP (median 30 mm, IQR 20–40 mm; Figure S4). Of women who responded to the survey, 9.6% (43/450) said that they would consider taking a drug every day for a 5 mm reduction in itch severity. Women’s responses did not differ depending on prior experience of ICP (median 30 mm, IQR 20–40 mm; Figure S5).

Clinicians estimated that a minimum of 35% (median, IQR 22.5–50%) of women would need to change from being unable to sleep through the night to being able to sleep through the night before they would consider prescribing UDCA. Women estimated that 50% (median, IQR 20–70%) of women would need to change status before they would consider taking the drug (Figure S6).

## Discussion

### Main findings

This planned secondary analysis of the PITCHES trial showed that in women with ICP, bile acid concentrations decreased after study enrolment in both the UDCA and placebo groups. Baseline bile acid concentrations did not affect a woman’s subsequent bile acid response to UDCA treatment.

A small decrease in itch score was found in women taking UDCA with a high baseline itch score, not seen in women with a low baseline itch score. The decrease was statistically significant but judged too small to be clinically meaningful for the majority of women and clinicians. As the test of interaction did not show significance, targeting treatment with UDCA for women with high baseline itch scores is not supported.

Further subgroup analyses evaluating baseline bile acid concentrations, itch scores and initial bile acid trajectories found no group where an effect of interaction was significant. This analysis therefore failed to identify any subgroup that might respond favourably to UDCA. A poor correlation was also demonstrated between bile acid concentrations and itch scores.

The results of the repeated surveys demonstrate that for both women and clinicians, their views on the size of a clinically meaningful reduction in itch severity were unchanged from 2011. Women and clinicians indicated that they deemed a median reduction in itch severity of 30 mm (on a visual analogue scale with a mean baseline itch of 60 mm) as clinically important.

### Strengths and limitations

A strength of this secondary analysis was the size of the original trial, which was considerably larger than any previous trial investigating the efficacy of UDCA in this population. The trial recruited women with a typical phenotype of ICP, with 24% of women having a baseline bile acid concentration ≥40 µmol/l, and the study findings are therefore likely to be generalisable to women with a similar clinical phenotype. The subgroups of baseline bile acid concentration analysed were based on clinically relevant thresholds that had previously demonstrated an association with differences in perinatal outcomes.^2^ Interaction tests were used to test for differences in treatment effect, which limited the possibility that the effect size seen in any individual subgroup was over‐interpreted. By understanding the size of a clinically meaningful reduction in itch severity through our survey results and from our patient co‐investigator, it was possible to consider whether or not any small differences identified were clinically relevant.

The original trial was not designed with a view to undertaking the present secondary analysis, and therefore no power calculation was undertaken to ensure that the study was sufficiently powered to find these effects. The study was limited by the size of certain subgroups, particularly when investigating women with baseline bile acid concentrations greater than 100 μmol/l (34 of 604 women). Although multiple analyses were undertaken, increasing the risk of false positive results, no significant differences were identified, mitigating the risk of false discovery.

### Interpretation (in light of other evidence)

The primary analysis of the PITCHES trial concluded that the routine use of UDCA in women with ICP should be reconsidered; in previously reported planned subgroup analyses evaluating the primary outcome (stillbirth, preterm delivery or neonatal unit admission) and its components, there was no significant interaction of highest baseline bile acid concentration (<40 μmol/l, ≥40 μmol/l), gestational age at randomisation (<34 weeks’ gestation, ≥34 weeks’ gestation), or pregnancy (singleton, twin).^6^ The secondary analysis presented here identified no subgroup of women in whom a reduction in bile acid concentration or itch score in response to treatment with UDCA was found. This result is in contrast with a previous, smaller, study by Glantz et al.^9^ that showed a reduction in maternal pruritus and bile acid concentration in a subgroup of women with bile acid concentrations ≥40 μmol/l after treatment with UDCA; however, the analysis was limited by its small size (12 women treated with UDCA versus 11 women treated with placebo) and by its duration (maximum treatment time 3 weeks).^9^ In contrast, this study included 116 women with baseline bile acid concentrations ≥40 μmol/l and an average treatment duration of over 4 weeks. The limited correlation between serum bile acid concentrations and severity of pruritus is consistent with other studies demonstrating that the likely pruritogens in ICP are progesterone sulphates and lysophosphatidic acid.^10^, ^11^

Reductions in mean bile acid concentrations were seen in all groups over the first few visits, regardless of whether the women were on active treatment or placebo. Previous clinical experience of this disease would have been confounded by almost universal treatment with UDCA, and the natural history of the disease may thus not be well understood. In particular, stratifying women by their initial bile acid trajectory demonstrated that a considerable proportion of women experienced a transient hypercholanaemia during pregnancy which resolved rapidly. This may represent a different pathology, e.g. secondary to a transient viral infection or exposure to a drug, from those with sustained hypercholanaemia in whom a diagnosis of ICP is more likely. In light of this, clinicians should take a detailed history and consider repeating bile acid measurements at subsequent visits to determine whether any elevation in bile acid concentration during pregnancy is persistent.

The repeat survey of women and clinicians found a high degree of concordance with the previous survey findings (2011),^8^ but some variability in the size of the itch reduction that was considered sufficient to justify taking a drug. The majority of women would only consider taking a drug if it yielded a reduction in itch severity of 30 mm, but a small proportion of women (9.6%) considered taking a drug for a reduction in itch score of only 5 mm. This variability may in part reflect different interpretations of what a given distance on the visual analogue scale represents.

## Conclusion

This analysis of women stratified by their baseline characteristics found no group in whom UDCA was effective in reducing bile acid concentrations or itch scores in a clinically important way. In the majority of women, mean bile acid concentrations decreased with time, regardless of treatment, emphasising the importance of evaluating the natural history of the clinical and laboratory investigations, in order to best identify women with sustained abnormal bile acid concentrations. Women and clinicians agree that a 30 mm reduction in itch severity on a 0–100 mm scale is clinically meaningful.

Previous work has implicated certain genetic mutations in the pathophysiology of ICP, including genes that directly influence biliary transport.^12^ It is possible that women with a genetic disposition to ICP due to defective biliary transport genes may respond to treatment differently from those without such mutations. Further research is needed both to identify the relevant genetic mutations and to determine whether affected women respond differently to treatment.

Quantification of bile acid concentrations is complex as UDCA itself is a bile acid and is included in standard laboratory measures of total bile acid concentration.^13^ There may be value in further research quantifying changes in harmful bile acid concentrations (such as cholic acid and chenodeoxycholic acid). Further work is needed to understand the pathophysiology behind pruritus in ICP, and develop an effective treatment for itching, in addition to targeting adverse perinatal outcomes associated with ICP.

Routine use of UDCA to reduce bile acid concentrations or itch scores should be reconsidered, and there is no justification for targeting women with high bile acid concentrations or high itch scores at presentation.

### Disclosure of interests

CW reports payments for advice from two pharmaceutical companies, Glaxo Smith Kline and Mirum Pharmaceuticals. JD reports grants from NIHR during the conduct of the study and grants from NIHR and Nutrinia outside the submitted work. JD was a member of the NIHR HTA General Board (from 2017 to 2018) and the NIHR HTA Maternity, Newborn and Child Health Panel (from 2013 to 2018). LCC reports grants from NIHR during the conduct of the study. All other authors declare no competing interests. Completed disclosure of interest forms are available to view online as supporting information.

### Contribution to authorship

LCC, CW, and JGT conceived the original trial. LCC, CW, PD, AS, EJ, JD, JGT and JC were involved in securing funding for the original trial. LCC and AS coordinated the original trial and data collection. PTS and JF did the secondary analyses, with input from LCC. JF wrote the article, with input from LCC. All authors reviewed, contributed to, and approved the final version of the manuscript.

### Details of ethics approval

The PITCHES trial was approved by the East of England—Essex Research Ethics Committee (15/EE/0010). Approval was granted on 18th February 2015.

### Funding

The trial was funded by the National Institute for Health Research Efficacy and Mechanism Evaluation Programme (Reference 12/164/16), following external peer review, including patient and public review. The funder had no role in study design, data collection and analysis, decision to publish, or preparation of the manuscript.

### Acknowledgements

LCC is funded by the National Institute for Health Research (NIHR) Professorship, RP‐2014–05–019. Paul T Seed is partly funded by King’s Health Partners Institute of Women and Children’s Health, Tommy’s (Registered charity no. 1060508) and by ARC South London (NIHR). The views expressed in this publication are those of the authors and not necessarily those of KHP, Tommy's, the NHS, the NIHR or the Department of Health. We thank the independent Trial Steering Committee (David Williams, Judith Hibbert, Julia Sanders, Deborah Stocken, Julian Walters, Win Tin) and the independent Data Monitoring Committee (John Norrie, William McGuire, Jenny Myers).

## Supporting information


**Figure S1.** Flow diagram of participants. Flow of participants prior to randomisation is described in the original PITCHES analysis (Chappell 2019).
**Figure S2.** Bile acid concentration stratified by initial bile acid trajectory in women with baseline bile acid concentration <40 and ≥40 μmol/l.
**Figure S3.** Itch score stratified by initial bile acid trajectory.
**Figure S4.** Results of survey question 1. Women included had prior experience of ICP.
**Figure S5.** Results of survey question 1 by previous experience of ICP.
**Figure S6.** Results of survey question 2. Women included had prior experience of ICP.

**Table S1.** Maternal baseline characteristics and outcomes for 527 women included in analysis.
**Table S2.** Perinatal outcomes for 558 infants born to 527 women included in analysis.
**Table S3.** Demographics of MCID survey respondents.
**Table S4.** Sensitivity analysis excluding women whose adherence to the intervention was less than 90% of trial medication taken, consistently reported.

**Appendix S1.** PITCHES secondary analysis statistical analysis plan (SAP).
**Appendix S2.** Minimal clinically important difference survey questions.

Supplementary Material

Supplementary Material

Supplementary Material

Supplementary Material

Supplementary Material

Supplementary Material

Supplementary Material

Supplementary Material

Supplementary Material

Supplementary Material

## Data Availability

The dataset will be available to appropriate academic parties on request from the Chief Investigator, Lucy Chappell, in accordance with the data sharing policies of King’s College London and the National Perinatal Epidemiology Unit Clinical Trials Unit, with input from the coinvestigator group where applicable, subject to submission of a suitable study protocol and analysis plan, on publication of all initial trial results.
