# Supplementary material for: Ursodeoxycholic acid in intrahepatic cholestasis of pregnancy: a secondary analysis of the PITCHES trial
Source: BJOG. 2020 Nov 8;128(6):1066–75. doi: 10.1111/1471-0528.16567 (PMC8246759; doi:10.1111/1471-0528.16567)
Supplement: Supplementary file 1 — Figure S1. Flow diagram of participants. Flow of participants prior to randomisation is described in the original PITCHES analysis (Chappell 2019). Figure S2. Bile acid concentration stratified by initial bile acid trajectory in women with baseline bile acid concentration <40 and ≥40 μmol/l. Figure S3. Itch score stratified by initial bile acid trajectory. Figure S4. Results of survey question 1. Women included had prior experience of ICP. Figure S5. Results of survey question 1 by previous experience of ICP. Figure S6. Results of survey question 2. Women included had prior experience of ICP. [file BJO-128-1066-s002.pdf]

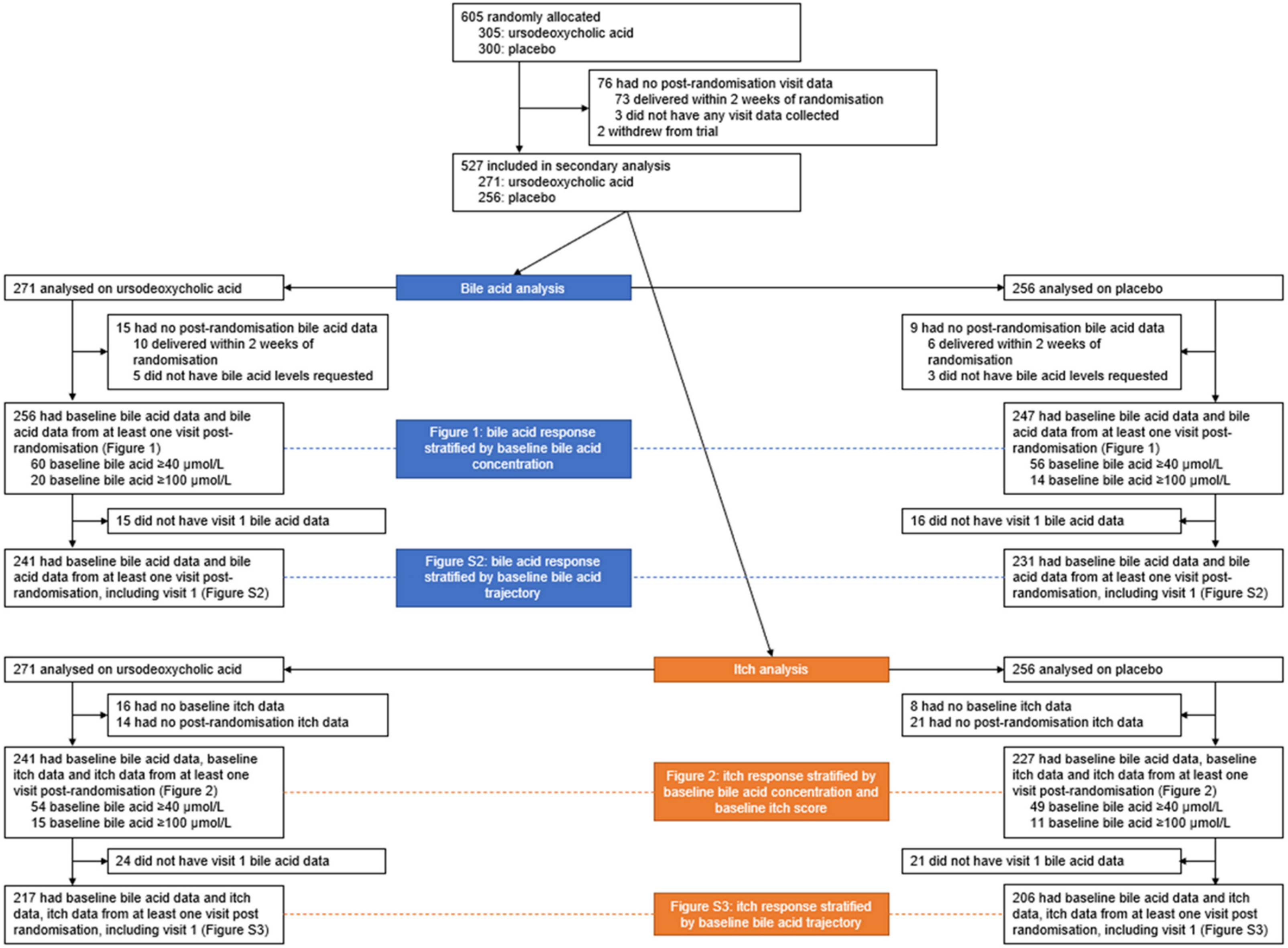

Figure S1. Flow diagram of participants. Flow of participants prior to randomisation is described in the original PITCHES analysis (Chappell 2019).

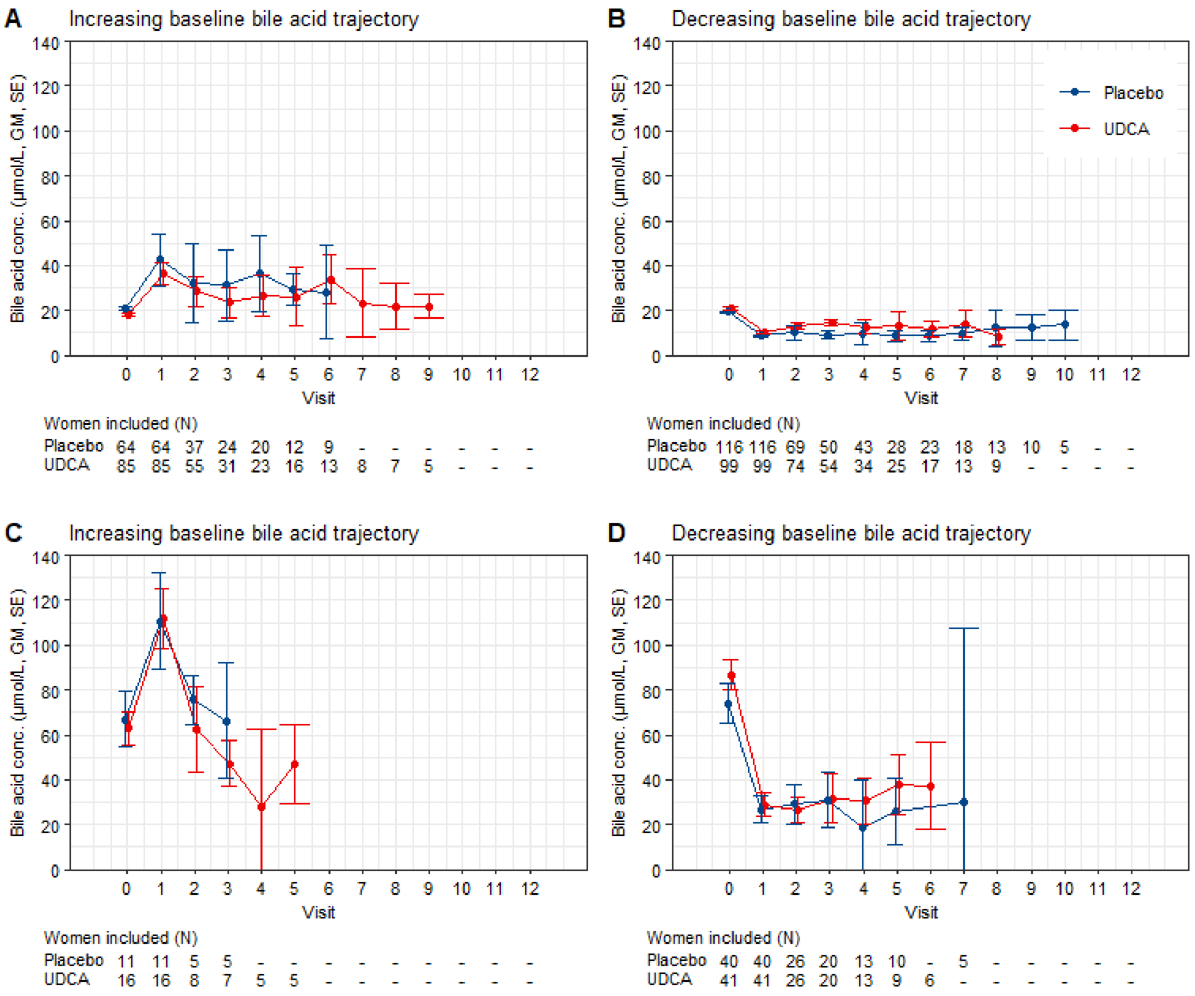

Figure S2. Bile acid concentration stratified by initial bile acid trajectory in women with baseline bile acid concentration <40 µmol/L and ≥40 µmol/L. Changes in bile acid concentration in women with baseline bile acid concentration <40 µmol/L, stratified by initial bile acid trajectory increasing (A) or decreasing (B) and in women with baseline bile acid concentrations ≥40 µmol/L, stratified by initial bile acid trajectory increasing (C) or decreasing (D) by visit post-randomisation. Numbers below each graph refer to number of women contributing results at each point. Only visits with five or more women were included. GM = geometric mean, SE = standard error.

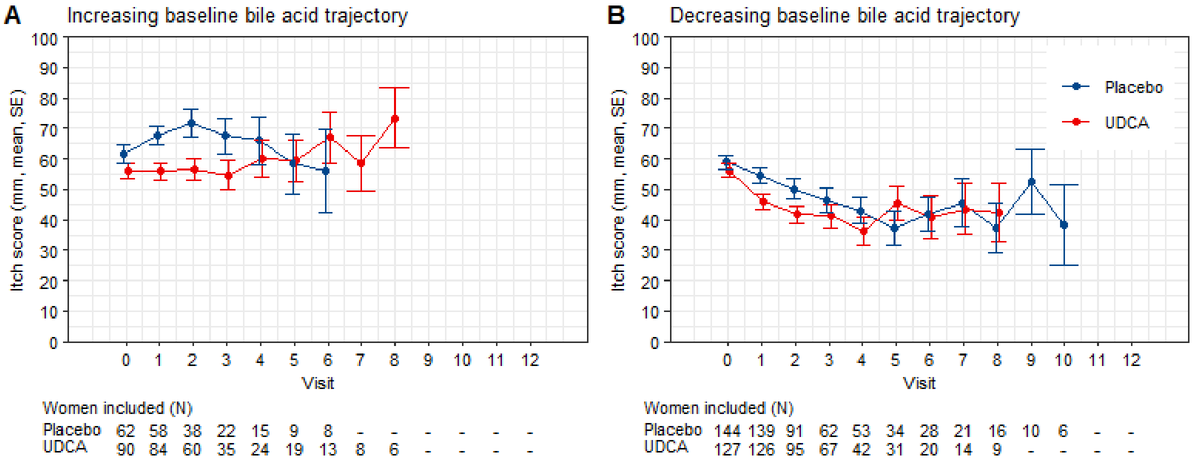

Figure S3. Itch score stratified by initial bile acid trajectory. Changes in itch score by visit post-randomisation, stratified by initial bile acid trajectory increasing (A) or decreasing (B). Numbers below each graph refer to number of women contributing results at each point. Only visits with five or more women were included. GM = geometric mean, SE = standard error.

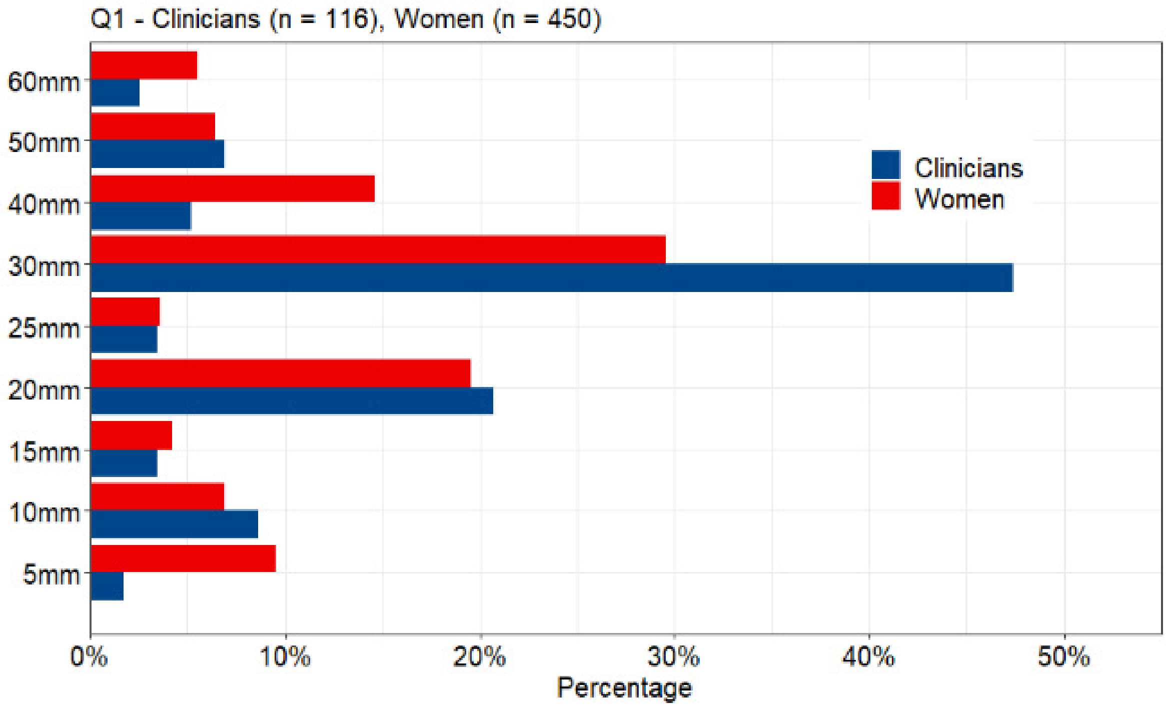

Figure S4. Results of survey question 1. Women included had prior experience of ICP.

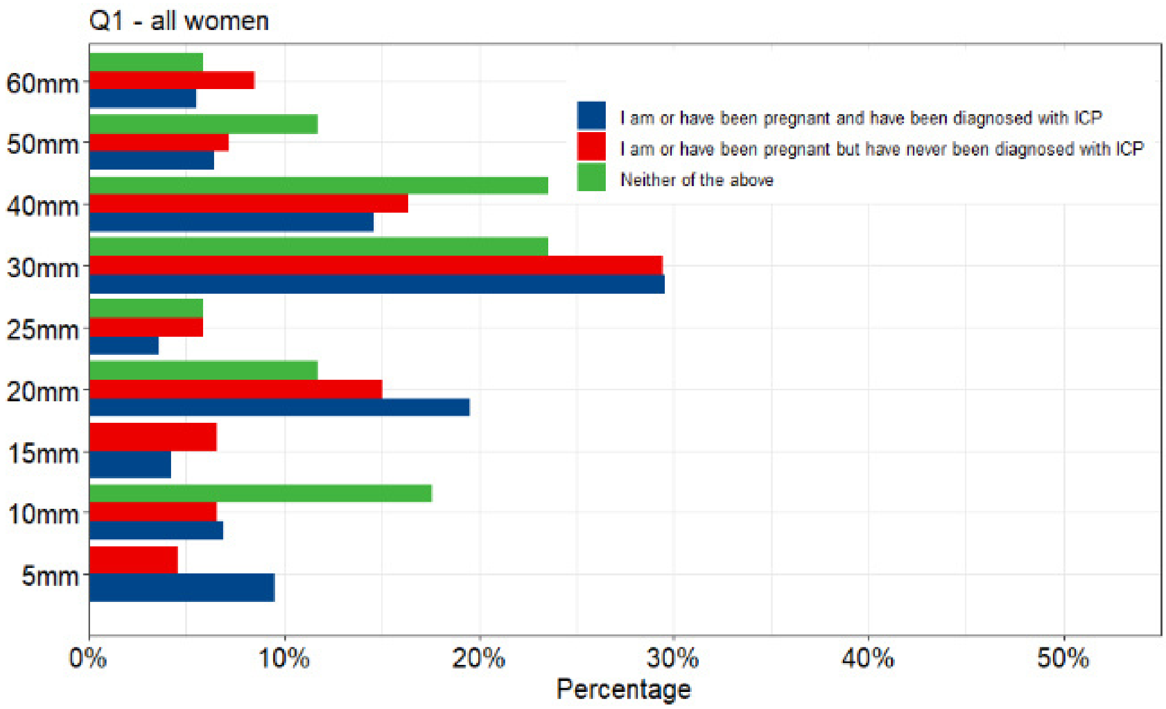

Figure S5. Results of survey question 1 by previous experience of ICP.

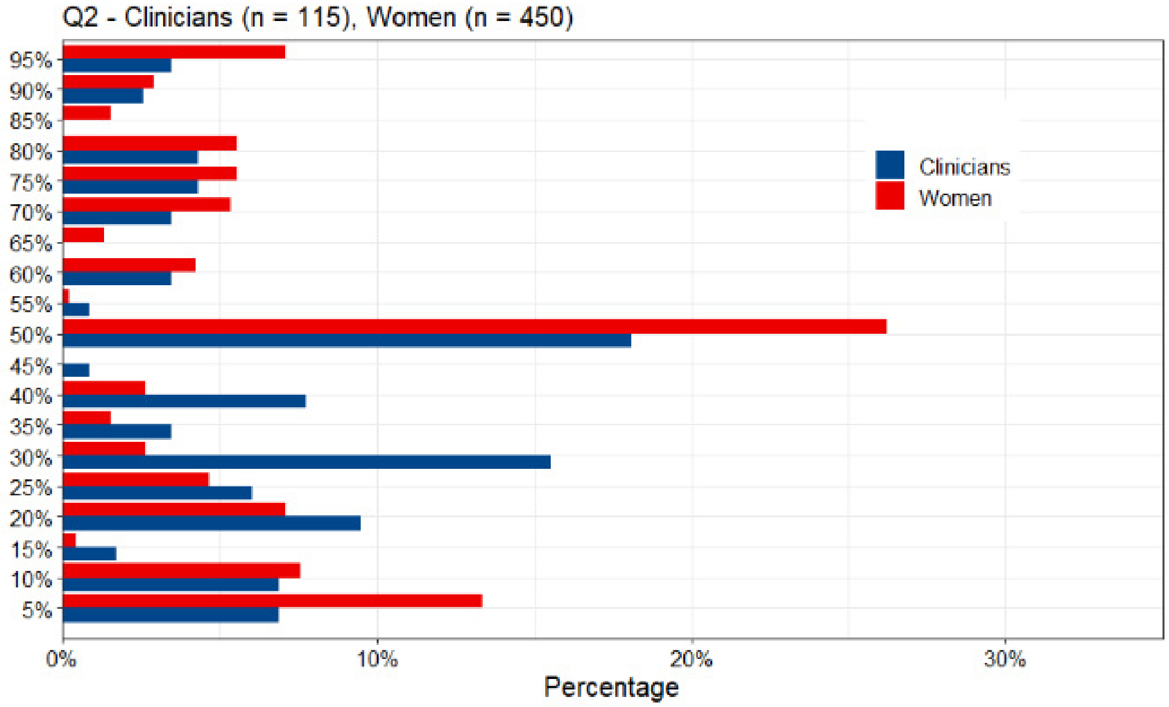

Figure S6. Results of survey question 2. Women included had prior experience of ICP.
