# Supplementary material for: Ursodeoxycholic acid in intrahepatic cholestasis of pregnancy: a secondary analysis of the PITCHES trial
Source: BJOG. 2020 Nov 8;128(6):1066–75. doi: 10.1111/1471-0528.16567 (PMC8246759; doi:10.1111/1471-0528.16567)
Supplement: Supplementary file 2 — Table S1. Maternal baseline characteristics and outcomes for 527 women included in analysis. Table S2. Perinatal outcomes for 558 infants born to 527 women included in analysis. Table S3. Demographics of MCID survey respondents. Table S4. Sensitivity analysis excluding women whose adherence to the intervention was less than 90% of trial medication taken, consistently reported. [file BJO-128-1066-s003.pdf]

Table S1. Maternal baseline characteristics and outcomes for 527 women included in analysis. Data are n (%), unless otherwise indicated; N is equal to the total number of participants in the group, unless otherwise indicated; <1% of observations are missing, unless indicated.

	Ursodeoxycholic acid (n = 271)	Placebo (n = 256)
**Baseline characteristics**		
Age, years	30.6 (5.7)	30.5 (5.2)
Ethnic group		
White	224 (82%)	212 (83%)
Black	8 (3%)	7 (3%)
Asian	28 (10%)	32 (13%)
Other	9 (3%)	5 (2%)
Not known	2 (1%)	0 (0%)
Body-mass index at start of pregnancy (kg/m²), mean (SD)	27.5 (6.5)	26.9 (6.2)
Previous pregnancy of ≥24 weeks' gestation	162 (60%)	169 (66%)
History of intrahepatic cholestasis of pregnancy, n/N (%)	87/159 (55%)	81/167 (49%)
Missing, n	3	2
Gestational age (weeks), median (IQR)	34.1 (31.9–35.4)	34.1 (30.7–35.6)
<34 weeks	130 (48%)	124 (48%)
34 to <37 weeks	125 (46%)	115 (57%)
≥37 weeks	16 (6%)	17 (7%)
Twin pregnancy	17 (6%)	14 (5%)
Itch score, mean (SD)	56.4 (24.7)	59.1 (25.2)
Highest baseline serum concentration before randomisation		
Bile acid (µmol/L), geometric mean (95% CI)	27.7 (25.5–30.1)	26.2 (24.1–28.3)
<40 µmol/L	209 (77%)	199 (78%)
≥40 µmol/L	62 (23%)	57 (22%)
Alanine transaminase, N	254	246
Alanine transaminase (U/L), geometric mean (95% CI)	68.7 (59.8–78.9)	56.6 (48.8–65.5)
**Outcomes**		
Maximum dose of trial medication		
One tablet once a day	4 (1%)	4 (2%)
One tablet twice a day	171 (63%)	158 (62%)
One tablet three times a day	61 (23%)	62 (24%)
Two tablets twice a day	35 (13%)	32 (13%)
Mode of onset of labour		
Spontaneous	27 (10%)	40 (16%)
Induced or pre-labour rupture of membranes and stimulation	194 (72%)	177 (69%)
Pre-labour caesarean	50 (18%)	39 (15%)
Estimated blood loss at delivery, mL	350 (250–600)	400 (250–600)
<500	175 (65%)	162 (63%)
≥500 and <1000	69 (25%)	65 (25%)
≥1000	27 (10%)	29 (11%)

Table S2. Perinatal outcomes for 558 infants born to 527 women included in analysis. Data are n (%), median (IQR), or mean (SD), unless otherwise indicated; N is equal to the total number of infants in the group, unless otherwise indicated; <1% of observations are missing, unless otherwise indicated.

	**Ursodeoxycholic acid (n = 288)**	**Placebo (n = 270)**
Perinatal death, preterm delivery or neonatal unit admission	63 (22%)	68 (25%)
In-utero fetal death	1 (<1%)	2 (1%)
Preterm delivery	46 (16%)	49 (18%)
Known neonatal death up to 7 days after birth	0 (0%)	0 (0%)
Neonatal unit admission for >4 h	38 (13%)	46 (17%)
Gestational age at delivery, weeks	37.6 (37.1–38.1)	37.4 (37.1–38.1)
Birthweight, g	3100 (2795–3392)	3055 (2740–3330)
Mode of delivery		
Spontaneous vaginal (cephalic)	174 (60%)	152 (56%)
Vaginal (breech)	1 (<1%)	3 (1%)
Assisted vaginal (cephalic)	18 (6%)	32 (12%)
Pre-labour caesarean	64 (22%)	54 (20%)
Caesarean	31 (11%)	29 (11%)
Presence of meconium-stained amniotic fluid	29 (10%)	46 (17%)

Table S3. Demographics of MCID survey respondents.

		All women (n = 650)
Experience	I am or have been pregnant and have been diagnosed with ICP	450 (73%)
	I am or have been pregnant but have never been diagnosed with ICP	153 (25%)
	Neither of the above	17 (3%)
		**Women with experience of ICP (n = 450)**
Age	18–24	26 (6%)
	25–34	228 (51%)
	35–44	176 (39%)
	45–54	17 (4%)
	55+	3 (1%)
		**Clinicians (n = 116)**
Age	18–24	1 (1%)
	25–34	16 (14%)
	35–44	48 (41%)
	45–54	39 (34%)
	55–64	11 (9%)
	65+	1 (1%)
Gender	Female	95 (82%)
	Male	20 (17%)
	Other	1 (1%)

Table S4. Sensitivity analysis excluding women whose adherence to the intervention was less than 90% of trial medication taken, consistently reported.

	UDCA (n = 205)	Placebo (n = 181)	Adjusted effects estimate (95% CI), p value	Interaction test
**Bile acid concentration, all women, N**	195	173		
Bile acid concentration (µmol/L), geometric mean (SD)	22.2 (2.3)	17.9 (2.7)	GMR 1.20 (1.01 to 1.42) p = 0.036	
**Baseline bile acid conc. <40 µmol/L, N**	152	135		p = 0.718
Bile acid concentration (µmol/L), geometric mean (SD)	19.3 (2.2)	15.6 (2.5)	GMR 1.20 (1.00 to 1.45) p = 0.055	
**Baseline bile acid conc. ≥40 µmol/L, N**	43	38		
Bile acid concentration (µmol/L), geometric mean (SD)	36.5 (2.0)	29.6 (2.9)	GMR 1.20 (0.81 to 1.77) p = 0.369	
**Baseline bile acid conc. <100 µmol/L, N**	183	166		p = 0.417
Bile acid concentration (µmol/L), geometric mean (SD)	21.3 (2.2)	17.2 (2.6)	GMR 1.22 (1.03 to 1.46) p = 0.025	
**Baseline bile acid conc. ≥100 µmol/L, N**	12	7		
Bile acid concentration (µmol/L), geometric mean (SD)	43.5 (2.3)	51.1 (2.6)	GMR 0.90 (0.40 to 2.01) p = 0.793	
**Itch score, all women, N**	191	168		
Itch score (mm), mean (SD)	49.7 (25.5)	55.6 (26.7)	MD -4.65 (-9.50 to 0.19) p = 0.060	
**Baseline itch score <60 mm, N**	86	67		p = 0.975
Itch score (mm), mean (SD)	40.7 (24.3)	44.9 (24.1)	MD -4.81 (-11.95 to 2.34) p = 0.189	
**Baseline itch score ≥60 mm, N**	105	101		
Itch score (mm), mean (SD)	57.2 (24.1)	62.6 (26.0)	MD -4.46 (-11.22 to 1.94) p = 0.168	
**Baseline bile acid conc. <40 µmol/L, N**	151	134		p = 0.668
Itch score (mm), mean (SD)	50.3 (26.0)	55.3 (26.5)	MD -4.05 (-9.46 to 1.35) p = 0.143	
**Baseline bile acid conc. ≥40 µmol/L, N**	40	34		
Itch score (mm), mean (SD)	47.6 (23.6)	56.5 (27.5)	MD -6.92 (-17.93 to 4.09) p = 0.222	
**Baseline bile acid conc. <100 µmol/L, N**	182	162		p = 0.257
Itch score (mm), mean (SD)	49.8 (25.6)	55.9 (26.4)	MD -5.08 (-9.93 to -0.22) p = 0.041	
**Baseline bile acid conc. ≥100 µmol/L, N**	9	6		
Itch score (mm), mean (SD)	49.1 (24.0)	47.5 (34.5)	MD -8.07 (-40.92 to 24.78) p = 0.639	
