# Supplementary material for: Ursodeoxycholic acid in intrahepatic cholestasis of pregnancy: a secondary analysis of the PITCHES trial
Source: BJOG. 2020 Nov 8;128(6):1066–75. doi: 10.1111/1471-0528.16567 (PMC8246759; doi:10.1111/1471-0528.16567)
Supplement: Supplementary file 3 — Appendix S1. PITCHES secondary analysis statistical analysis plan (SAP). Appendix S2. Minimal clinically important difference survey questions. [file BJO-128-1066-s013.pdf]

Appendix S1. PITCHES secondary analysis statistical analysis plan (SAP).

1 Does baseline bile acid concentration or baseline itch score moderate a woman's bile acid concentration or itch score response to treatment with UDCA?

Subgroups to be analysed:

a. Bile acid response by baseline bile acid concentration (≥40 µmol/L vs <40 µmol/L and ≥100 µmol/L vs <100 µmol/L)
b. Itch response by baseline bile acid concentration (≥40 µmol/L vs <40 µmol/L and ≥100 µmol/L vs <100 µmol/L)
c. Itch response by baseline itch score (≥60 mm vs <60 mm)

Subgroups will be formed based on a specific baseline characteristic. Averages of all available post-randomisation observations will be reported and compared using an appropriate comparator.

As bile acid concentrations demonstrate a lognormal distribution, a geometric mean will be used to calculate each participant's average post-randomisation bile acid concentration. The subgroups will be compared using a geometric mean ratio.

An arithmetic mean will be used to calculate each participant's mean post-randomisation itch score. The subgroups will be compared using a mean difference.[1]

An interaction test (likelihood ratio version) will be used to test for a difference in treatment effect between the individual subgroups.

The trajectory of the treatment effect for each subgroup will be visualised by plotting average bile acid concentration and average itch score by visit.

The analysis of other baseline characteristics will also be considered:

i. Gestation at randomisation (<34 vs 34-37 vs >37)
ii. History of ICP
iii. Baseline ALT ≥70
iv. Ethnicity

2 Do maternal itch scores correlate with bile acid concentration?

All available pairs of itch scores and bile acid concentrations will be plotted for all participants at all time points. A linear model will be used to determine the degree of correlation between the two.

3 Can particular patterns of response (to treatment and to placebo) be demonstrated?

Patterns of change in bile acid concentrations in response to the intervention (both placebo and active treatment) will be classified as responders and sustained responders.

Responders will be defined as women who show a reduction in bile acid concentration between the pre-randomisation sample and the first measured bile acid after randomisation (up to visit 3).

Sustained responders will be defined as women who's geometric mean of the log of the bile acid concentration is less than the pre-randomisation bile acid concentration.

Individual women will then be coded as responders (1/0) and sustained responders (1/0). These two variables will be combined to generate four possible patterns of response.

i. No response (0,0)
ii. Initial response (1,0)
iii. Late response (0,1)
iv. Sustained response (1,1)

A similar analysis will be performed on itch scores.

Notes:

1. We considered looking at a category difference of -10 mm, -20 mm, etc to investigate itch response but we decided that we needed to look for a subgroup that had a mean difference greater than the minimally clinically important difference (MCID) as otherwise information is lost about women who get worse. Any increase in women with a reduction of -20 mm may be balanced by a group of women who see an increase in their itch score of the same amount.

As we were unable to identify any subgroup of women based on their baseline bile acid concentration and itch scores in whom UDCA was effective, we did not continue with our prespecified plan to define different patterns of response to treatment and placebo. We also did not believe it was appropriate to extend the analysis to other baseline characteristics.

Appendix S2. Minimal clinically important difference survey questions.

Clinician ICP survey

We are planning to perform a secondary analysis of the PITCHES trial data (UDCA vs. placebo in women with ICP). As in our primary analysis, we are using maternal itch scoring as an outcome measure. We previously surveyed clinicians to determine a clinically useful decrease in itching and we are now repeating this survey prior to our secondary analysis. This survey contains two questions about the effect that the drug would need to have for you to consider recommending it to a woman.

1. We assessed maternal itching using a Visual Analogue Scale where 0mm represented no itching and 100mm the worst itching. You will be aware that UDCA is currently not licensed for use in pregnancy and that there is no definitive evidence of fetal benefits or harms. We would like to pre-specify a CLINICALLY USEFUL decrease in itching score so that we do not over-interpret or misinterpret our results – which may show some statistically significant, but not clinically meaningful difference. We are also asking the same question to women who have had ICP.

Imagine you are advising a patient whose worst episode of itching in the previous 24 hours was 60mm. What decrease in itching score do you think is clinically meaningful, such that you would recommend UDCA to her?

- 5mm (a very small decrease in itching)
- 10mm
- 15mm
- 20mm
- 25mm
- 30mm
- 40mm
- 50mm
- 60mm (complete resolution of itching)

2. Imagine you are treating women with itching that is so severe that they cannot sleep at night. Suppose UDCA will reduce SOME of these women's itch to a level at which they can sleep at night.

What percentage of women would need to have their itching reduced to this extent (from not being able to sleep at night to being able to sleep at night) for you to recommend UDCA to them?

- 5% (One out of twenty women are able to sleep at night. Nineteen out of twenty women still have itching so severe that they cannot sleep.)
- 10%
- 15%
- 20%
- 25%
- 30%
- 35%
- 40%
- 45%
- 50%
- 55%
- 60%

- 65%
- 70%
- 75%
- 80%
- 85%
- 90%
- 95% (Nineteen out of twenty women are able to sleep at night. One out of twenty women still have itching so severe that they cannot sleep.)

3. How old are you?

- Under 18
- 18-24
- 25-34
- 35-44
- 45-54
- 55-64
- 65+
- Prefer not to say

4. What is your gender?

- Female
- Male
- Other (specify)

Women ICP survey

We would like you to imagine that you are pregnant and have intrahepatic cholestasis of pregnancy (ICP). You have been offered a drug that may help reduce your itching. This drug has been tested in pregnancy and it seems to be safe for both the mother and the baby. It is also known to be safe for people who aren't pregnant. We would like to understand the effect that the drug would need to have for you to consider taking it.

1. We would like you to imagine that you agree to take the drug and that every week you will be asked to score your itch on a scale from 0 (no itching) to 100 (the worst itching). Before taking this drug your score is 60.

By how much would you need to see your itching score decrease for you to feel that the drug is worth taking?

- 5 (a very small decrease in itching)
- 10
- 15
- 20
- 25
- 30
- 40
- 50

- 60 (complete resolution of itching)

2. We would like you to imagine that you are suffering from itching that is so severe you cannot sleep at night. You are told that this new drug will reduce itching to the point that some (but not all) women will be able to sleep.

In what percentage of women would the drug need to work to this degree for you to consider that it is worth taking?

- 5% (One out of twenty women are able to sleep at night. Nineteen out of twenty women still have itching so severe that they cannot sleep.)
- 10%
- 15%
- 20%
- 25%
- 30%
- 35%
- 40%
- 45%
- 50% (Ten out of twenty women are able to sleep at night. Ten out of twenty women still have itching so severe that they cannot sleep.)
- 55%
- 60%
- 65%
- 70%
- 75%
- 80%
- 85%
- 90%
- 95% (Nineteen out of twenty women are able to sleep at night. One out of twenty women still have itching so severe that they cannot sleep.)

3. Please tick the statement below that applies to you:

- I am or have been pregnant and have been diagnosed with ICP
- I am or have been pregnant but have never been diagnosed with ICP
- Neither of the above

4. How old are you?

- Under 18
- 18-24
- 25-34
- 35-44
- 45-54
- 55+
- Prefer not to say
